# Bioluminescent Imaging and Histopathologic Characterization of WEEV Neuroinvasion in Outbred CD-1 Mice

**DOI:** 10.1371/journal.pone.0053462

**Published:** 2013-01-02

**Authors:** Aaron T. Phillips, Charles B. Stauft, Tawfik A. Aboellail, Ann M. Toth, Donald L. Jarvis, Ann M. Powers, Ken E. Olson

**Affiliations:** 1 Department of Microbiology, Immunology, and Pathology, Colorado State University, Fort Collins, Colorado, United States of America; 2 Department of Molecular Biology, University of Wyoming, Laramie, Wyoming, United States of America; 3 Division of Vector-Borne Infectious Diseases, Centers for Disease Control, Fort Collins, Colorado, United States of America; University of Texas Medical Branch, United States of America

## Abstract

Western equine encephalitis virus (WEEV; *Alphavirus*) is a mosquito-borne virus that can cause severe encephalitis in humans and equids. Previous studies have shown that intranasal infection of outbred CD-1 mice with the WEEV McMillan (McM) strain result in high mortality within 4 days of infection. Here *in vivo* and *ex vivo* bioluminescence (BLM) imaging was applied on mice intranasally infected with a recombinant McM virus expressing firefly luciferase (FLUC) to track viral neuroinvasion by FLUC detection and determine any correlation between BLM and viral titer. Immunological markers of disease (MCP-1 and IP-10) were measured and compared to wild type virus infection. Histopathology was guided by corresponding BLM images, and showed that neuroinvasion occurred primarily through cranial nerves, mainly in the olfactory tract. Olfactory bulb neurons were initially infected with subsequent spread of the infection into different regions of the brain. WEEV distribution was confirmed by immunohistochemistry as having marked neuronal infection but very few infected glial cells. Axons displayed infection patterns consistent with viral dissemination along the neuronal axis. The trigeminal nerve served as an additional route of neuroinvasion showing significant FLUC expression within the brainstem. The recombinant virus WEEV.McM.FLUC had attenuated replication kinetics and induced a weaker immunological response than WEEV.McM but produced comparable pathologies. Immunohistochemistry staining for FLUC and WEEV antigen showed that transgene expression was present in all areas of the CNS where virus was observed. BLM provides a quantifiable measure of alphaviral neural disease progression and a method for evaluating antiviral strategies.

## Introduction

Of the 29 mosquito-borne viral species within the *Alphavirus* genus (*Togaviridae*), at least 16 are known to cause disease in humans and animals [1]–[4]. Although arthritis, acute flu-like illness, and rash are attributable to many alphaviral infections, some alphavirus species lead to CNS infection and encephalitis. Alphaviruses most often associated with CNS infection are limited to the Americas, and include strains of eastern equine encephalitis virus (EEEV), Venezuelan equine encephalitis virus (VEEV), and western equine encephalitis virus (WEEV). WEEV is normally maintained in a transmission cycle involving *Culex tarsalis* mosquitoes and passerine birds [5]. Equids and humans can be infected but do not contribute to the maintenance cycle. WEEV was first isolated from an outbreak of equine encephalitis in the San Joaquin Valley of California that affected almost 6,000 horses and was associated with an equine mortality rate of 50% [6]. Enzootic activity of WEEV is detected most summers in southern California via sero-conversion of sentinel animals or testing pools of primary vectors [7]. According to the USDA, epizootics have been reported in horses (Canada 1975), turkeys (California 1993–1994; Nebraska 1957), and emus (Texas and Oklahoma 1992). These findings highlight the potential for a WEEV epidemic outbreak in humans. Naturally acquired infection of humans has been estimated to yield fatality rates of 8% to 15% [8]. Human patients may present clinically with symptoms ranging from an acute febrile illness to fulminant encephalitis. Neurologic sequelae may be present in survivors, particularly children and infants [9]. Experimental evidence suggests that WEEV strains can be categorized into high and low mortality phenotypes in mice [10], [11]. Among the high mortality phenotypes, McM induces rapid and lethal encephalitic disease in a mouse infection model and is the basis of the data reported here.

New World alphavirus strains readily cause encephalitis after aerosol or intranasal exposure in animal models making these alphaviruses potential biodefense agents requiring efficacious therapeutic and vaccine-based responses. Previous studies have shown that, following respiratory routes of inoculation, neuroinvasion occurs preferentially through the olfactory tract by initial infection of neuroepithelia [12]–[14]. Responsible for sensing odorants, neuroepithelial tissue is in direct contact with the environment and easily subject to initial infection by these routes. Viral dissemination into the CNS likely occurs through the long axonal projections of olfactory sensory neurons (OSN), which converge upon the olfactory bulb of the CNS (Figure S1). Histological evidence supports this proposed mechanism [14] however, published characterizations are few. Notably, reports characterizing WEEV infection in an animal model are rare [15]. Additionally, WEEV is a naturally-occurring recombinant virus generated from ancestral EEEV- and Sindbis- like virus [16]. As EEEV and Sindbis have markedly different disease phenotypes in humans, a better description of WEEV pathogenesis is needed to identify infection patterns.

All alphaviruses have an enveloped nucleocapsid containing a single-stranded, positive-sense RNA genome with a 5′ methylated cap and 3′ polyadenylated termini. The 5′ end of the viral genome is translated into 4 nonstructural proteins (nsP 1–4) that form viral replication complexes. A negative-strand RNA replication intermediate is generated and contains a subgenomic promoter (SGP) or internal initiation site that initiates transcription of the 26S subgenomic RNA. The 26S subgenomic RNA encodes the structural proteins (Capsid, E3, E2, 6K, and E1) used in the assembly of new virions. Genomic RNA is fully infectious when transfected into permissive cells. Consequently, alphaviruses are readily manipulated in the laboratory using traditional cloning techniques followed by *in vitro* transcription of the viral genome. Recombinant alphaviruses have been developed in which the SGP sequence is duplicated to drive expression of heterologous genes during infection [17]–[26]. The infectious cDNA clones are sometimes referred to as alphavirus expression systems (AES) [19]. Most AESs which have been developed to date have been based on Old World alphaviruses such as Sindbis [17], [18], [20], [22]–[24], [27]–[30], Semliki Forest [31], O'nyong-nyong [32], or chikungunya viruses [26]. Although these AESs have significantly enhanced our understanding of virus-vector and virus-host interactions, reports of AESs based on propagating New World encephalitic alphavirus isolates are less prevalent in the literature [21], [33], [34]. To our knowledge, there have been no reports describing an infectious WEEV-based AES.

A more complete understanding of the alphavirus infection patterns in vertebrates is crucial to characterizing the pathogen-host relationship. The technology of *in vivo* imaging promises to streamline the process of investigating infectious agents in an animal model. Firefly luciferase (FLUC) is a commonly used in vivo BLM reporter. FLUC catalyzes the oxidation of its substrate, luciferyl adenylate (luciferin), with the net products being light, in the form of a photon, and oxyluciferin [35]. Firefly luciferase (FLUC) and its substrate, luciferin, was first used to describe the distribution of bacteria in a living host [36] and has subsequently been used to describe infection in mice for herpesvirus type-I [37], a neurovirulent strain of Sindbis virus [17], [23], [24], [38], VEEV [21], EEEV [33], [38], and human immunodeficiency virus (HIV) gene expression [39]. Research applying *in vivo* imaging technology to analyze vaccinia virus infection has shown potential for predicting lethality of virus infection based on luminescence [40].

A well-characterized animal model is a crucial component of antiviral research. This is especially true when considering pathogens such as WEEV in which the target cells (neurons) are difficult to work with in culture. Additionally, components of the CNS, such as the blood-brain-barrier and multiple cell types are absent from *in vitro* model systems. Outbred CD-1 mouse model of McM infection has been developed and used to characterize the infection using traditional methods [41]. Chemokines such as MCP-1 and interferon-gamma increased significantly in infected brain tissue [41]. The inflammatory process plays a role in neuropathogenesis, but this role may be secondary to neuronal death resulting directly from viral replication. This is supported in studies with VEEV which have shown extended mean time to death of mice treated with anti-thymocyte serum [42]. In the case of neurovirulent Sindbis virus, neuronal death is due to inflammatory and excitotoxic insults or apoptosis depending on the strain of virus, age of the mouse model, and specific neuroanatomical location [43].

The goals of these studies were two-fold. First, we sought to better characterize the process of neuroinvasion and CNS dissemination in the mouse model and use BLM imaging to follow infection and identify sub-anatomic regions where the virus attains high levels of replication. The second goal of these studies was to characterize the luciferase-expressing recombinant WEEV for its ability to induce disease similar to the wild-type virus, and to determine if BLM signal correlated with biological markers of disease. Ultimately, quantitative measurements of viral BLM can be used to evaluate the efficacy of antiviral strategies within the intact live animal. We show that the McM-based AES is capable of producing a conveniently measured marker of infection and, in doing so, provides a system for evaluating therapies aimed at preventing disease arising from inhalation of New World alphavirus strains. Currently, there are no approved therapies for use in humans, and the WEEV AES presented in this report should enhance the utility of the mouse model in developing effective treatments.

## Materials and Methods

### Virus Construction

A full-length infectious clone (IC) of the McMillan strain of WEEV (pMcM) was a kind gift of Dr. Thomas Welte (Colorado State University), was derived from virus obtained from the Arbovirus Reference Collection at the Center for Disease Control and Prevention in Fort Collins, CO, USA, and has been previously studied [11]. Detailed descriptions of the molecular cloning methods used to engineer WEEV.McM.FLUC are provided in supplemental materials and methods section (Methods S1). In brief, SGP sequence (nucleotides 7341–7500 of viral genome) was duplicated immediately downstream of the last nucleotide of E1. FLUC was inserted immediately downstream of the new SGP. The duplicated SGP was used to initiate transcription of FLUC and the 2^nd^ subgenomic mRNA.

### Rescue of Virus from Infectious Clone

Linearized ICs were purified by QIAprep Spin MiniPrep Kit (Qiagen, Valencia, CA USA) and IC genomic RNA was *in vitro* transcribed using a T7 RNA Polymerase and MAXIscript™ kit (Life Technologies, Grand Island, NY USA). BHK cells (2×10^7^ cells in 400 µL) were electroporated with 20 µL of genomic RNA using an ECM 630 electroporator (BTX Harvard Apparatus, Holliston, MA USA). Two pulses of 450 V, 1200 Ω, and 150 µF were administered. Media was taken from electroporated cells and passaged once in BHK cells to make a stock virus. Supernatant was collected at 48 hpi and stored at −80°C. This stock was quantified using plaque titration in Vero cells and used for subsequent experiments. We have previously observed WEEV-based double subgenomic constructs expressing FLUC to be acceptably stable. Substantial luciferase activity was present out to the 3^rd^ passage in BHK-21 cells and out to the 4^th^ passage in C6/36 cells (data not shown).

### Plaque Titrations

Virus titrations were performed in duplicate and plaque assays were performed as described by Liu et al. (1970).

### Mouse Infection and Imaging

All animal protocols used in these experiments were reviewed and approved by the Animal Care and Use Committee at Colorado State University (Permit #11-2605A). Mice were handled in compliance with the PHS Policy and Guide for the Care and Use of Laboratory Animals. Female 4–5 week old CD-1 mice (Charles River Labs, Wilmington, MA USA) were used in this study. Intranasal inoculation was conducted at a dose of 1×10^4^ PFU of McM or WEEV.McM.FLUC in a volume of 20 µL delivered drop wise onto the nostrils of lightly anesthetized animals. Imaging was performed after 150 mg/kg of luciferin (30 mg/mL stock diluted in PBS) was injected subcutaneously dorsal to the cervical spine of each infected animal. Administration of luciferin via this route has been shown to result in more consistent signal compared to intraperitoneal administration [44]. Each animal was imaged 10–15 minutes after injection of substrate. Uninfected mice were used as an imaging control to adjust for background. Mice were anesthetized by administration of isoflurane (Minrad Inc, Bethlehem, PA USA) through an XGI-8 anesthesia system (Caliper Life Sciences) connected to the IVIS 200 camera during imaging. Exposure time was kept to 3 minutes under standard settings for the camera. Living Image 3.0 software (Caliper Life Science) was used to analyze and process images taken using the IVIS 200 camera. A threshold for significant BLM was established using negative imaging controls at 5×10^3^ p/s/cm^2^/sr. Total light emission from each mouse was accomplished by creating a region of interest of standard size for each mouse and collecting light emission data using the software.

Sagittal whole head sections of infected mice were imaged by injecting mice with 150 mg/kg of luciferin (30 mg/mL stock diluted in PBS) 24 h, 48 h, 60 h, and 72 h post-infection. After 10 minutes, mice were injected with another dose of luciferin, and promptly euthanized via inhalation of a lethal dose of isoflurane. Animals were decapitated and whole heads bisected along the medial sagittal plane. Resulting sections were briefly rinsed with PBS and promptly imaged.

### Chemokine Quantification

Three animals were euthanized at each of three time points (24, 48, and 72 h.p.i.) after obtaining BLM images. Brains were harvested and assayed as previously described [41]. Briefly, whole brains were harvested, homogenized in buffered media, and clarified by centrifugation. Supernatant was collected and divided into aliquots. Single aliquots were used to assay for immunological markers (MCP-1 and IP-10, R&D Systems) or virus quantification by plaque assays as described above.

### Immunohistochemistry

Paraffin -embedded formalin fixed tissue was rehydrated, treated with Tris-EDTA pH 9.0 at 90°C for 15 minutes, and blocked with SuperBlock T20 (Thermo, Rockford, IL). Biotinylated polyclonal rabbit anti-FLUC antibody (Abcam, Cambridge, MA) was used at 1∶1000 dilution and incubated overnight at 4°C. Primary antibody was washed 3 times with Tris-buffered saline containing 0.03% Tween 20(TBST). Secondary antibody was strepavidin-horseradish peroxidase (Rockland, Gilbertsville, PA) and was used at a 1∶6000 dilution and incubated for 30 minutes at room temperature. Slides were again washed three times with TBST. 3,3'-diaminobenzidine (DAB) was added to the slides and allowed to develop stain for 5 minutes. Hematoxylin was used to counterstain. Hyperimmune horse serum generated against WEEV Fleming strain (CDC, Fort Collins, CO) was used for anti-WEEV IHC at 1∶600 dilution. Secondary antibody was HRP-conjugated rabbit polyclonal antibody to horse IgG heavy and light chain (Abcam, Cambridge, MA) used at a 1∶3500 dilution. All other conditions remained unchanged.

### Preparation and Administration of Vaccine

Cationic liposomes (100 mM DOTIM lipid+cholesterol) in 10% sucrose solution were provided by Dr. Steve Dow (CSU). Antigen was produced in baculovirus expression systems and purified similarly to that previously described [45]. Briefly, antigen (WEEV E1 glycoprotein ectodomain) was produced in Sf9 cells using a recombinant baculovirus encoding the first 408 amino acids of WEEV McM E1 fused to a C-terminal 8XHis tag. The E1 ectodomain protein was purified from cell culture supernatant by nickel affinity chromatography. Cationic-liposome–nucleic-acid-complexes (CLNCs) were prepared as previously described [41]. Briefly, liposomes were diluted 1∶5 in sterile Tris-buffered 5% dextrose water (pH 7.4). Poly (I:C) (InvivoGen, San Diego, CA) was then added to a final concentration of 0.1 mg/ml causing spontaneous formation of CLNCs. The formed complexes were mixed with WEEV E1 glycoprotein ectodomain antigen to a final concentration of 50 µg/mL. Resulting liposome-antigen-nucleic acid-complexes were used to vaccinate mice at 28 days (prime) prior to challenge and again at 14 days (boost) prior to challenge. Untreated control animals received only CLNCs and a sham antigen preparation. Additional controls were untreated mice that were not inoculated (to control for background luminescence). Animals were administered luciferin as described in previous section. There were a total of 3 mice per group.

### Statistics

All titration data were log_10_ transformed and compared using unpaired Student’s *t* test. In determining the correlation of PFU with BLM, curves were analyzed using Pearson correlation with 95% confidence interval. For chemokine quantification comparisons, unpaired t test was used. Analysis was conducted using statistical analysis software (SAS) version 9.2. Survival curves were subjected to Kaplan-Meier (log rank test) analysis using Prism version 4.00 for Windows (GraphPad). Quantitative analysis of bioluminescence in the assessment of vaccine efficacy was conducted using two-tailed *t*-test.

## Results

### Recombinant FLUC-expressing WEEV Phenotype in CD-1 Mice

**Figure 1:**
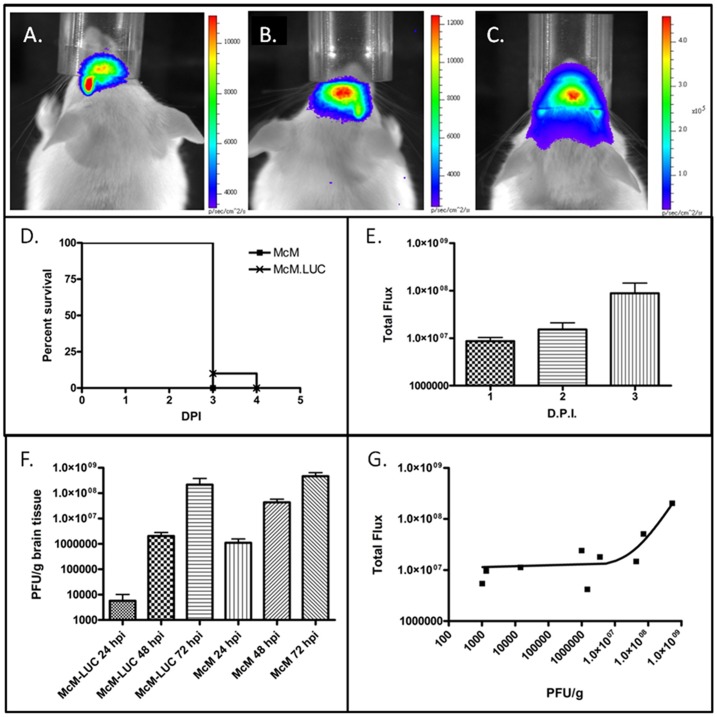
*In vivo* BLM imaging of infection progress using WEEV.McM.FLUC. A: 24 hpi B: 48 hpi C: 72 hpi D: Survival analysis of WEEV.McM.FLUC and wild-type virus (WEEV.McM) from subcutaneous and intranasal virus challenge experiments. Note uniform lethality resulting from intranasal exposure by WEEV.McM.FLUC. E: FLUC activity was quantitatively measured in each animal at 1, 2, and 3 d.p.i. time points. Results from BLM analysis demonstrate robust FLUC activity as infection progressed with the greatest increase observed between days 2 and 3 post-infection. F: Brains of animals infected with WEEV.McM attain a higher viral titer more rapidly when compared with WEEV.McM.FLUC. WEEV.McM.FLUC titers approach that of McM by 72 h.p.i. G: Regression analysis of viral titer versus FLUC activity. Linear regression line appears curved due to log10 scaling of axis as required to clearly depict all data points (R^2^ = 0.9807).

WEEV.McM.FLUC infection of CD-1 mice was characterized after administering virus by the intranasal route. WEEV.McM.FLUC virus expressed FLUC throughout infection (Figure 1A–C) where signal was restricted to the head. To determine if FLUC signal in other anatomical regions was potentially masked by signal from the head, mice showing signs of disease and strong luciferase signal in the head region were euthanized, decapitated, and imaged again with an opened visceral cavity. No signal was detected outside the head region (data not shown). Exponential increases in BLM signal (photons/second/centimeter^2^/steradian) were observed from day 2 to day 3 post-inoculation (Figures 1B, C, & E). Infection progressed from the nasal cavity toward more caudal regions and was symmetrical with respect to the sagittal axis. A 0% survival rate was observed for both WEEV.McM and WEEV.McM.FLUC in animals (n = 10) inoculated by the intranasal route and a comparison of mouse survival showed no significant difference between the two viruses (Figure 1D) (*P* value = 0.4795). We then compared FLUC activity against measured infectious virus titers from whole brain homogenates (Figure 1E–G). WEEV.McM virus replicated to 100- fold PFU/mL higher titer than WEEV.McM.FLUC within the first 24 hpi. WEEV.McM.FLUC virus titer was statistically similar to WEEV.McM virus titers by 72 hpi. Comparison of total flux (p/s) for each WEEV.McM.FLUC -inoculated mouse with viral titer measured within the whole brain (Figure 1G), showed a strong correlation (Pearson R = 0.9903 and R^2^ = 0.9807). As expected, uninfected control animals receiving daily luciferin injections did not show any signs of disease.

### Localization of Virus by *ex vivo* Imaging of Medial Sagittal Cross-section

**Figure 2:**
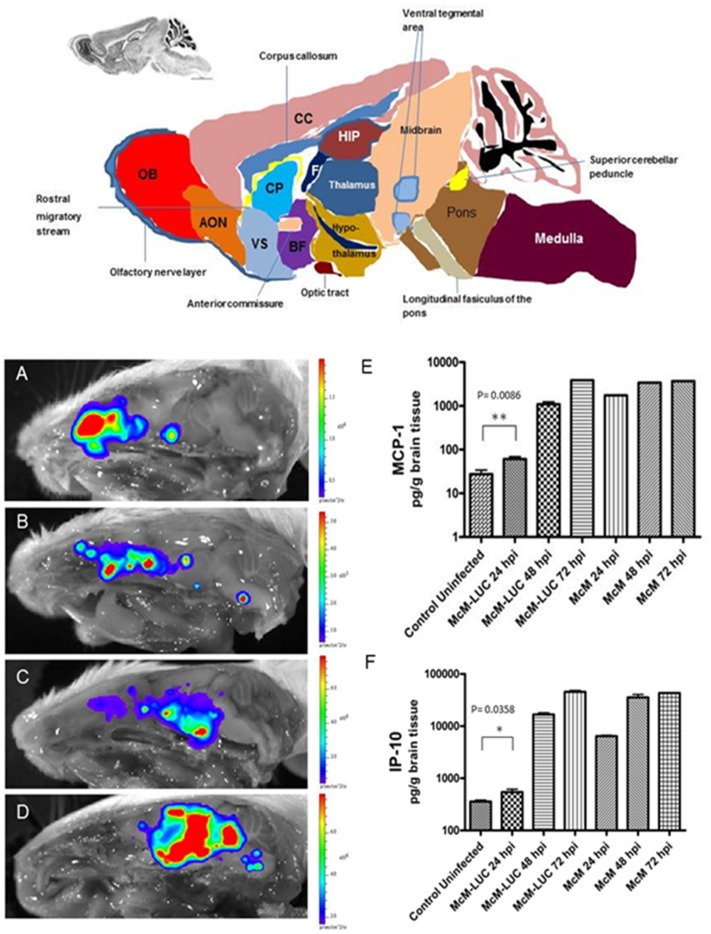
Schematic depiction of anatomical organization of mouse brain in medial sagittal view. AON: anterior olfactory nucleus, BF: basal forebrain, CC: cerebral cortex (isocortex), CP: caudate putamen, F: fornix, HIP: hippocampus, OB: olfactory bulb, VS: ventral striatum. Progress of infection with WEEV after intranasal inoculation (A–D). Whole heads were bisected along sagittal midline and imaged at 24 hpi (A), 48 hpi (B), 60 hpi (C) and 72 hpi (D). Luciferase activity pattern is consistent with dissemination along olfactory pathways. Regions consistent with initial infection of the nasal turbinates show pronounced FLUC activity at 24 hpi. However, nasal turbinate BLM activity is exceeded by signal from areas consistent with infection proceeding through olfactory information processing within the CNS, including the lateral olfactory tract, anterior olfactory nucleus, basal ganglia, thalamus, and cerebrum. Immunological markers of disease (MCP-1 and IP-10), resulting from WEEV.MCM.FLUC, are strongly induced and comparable to WEEV.McM at 3 d.p.i. (E–F).

Neuroinvasion and CNS dissemination *in situ* was detected in CD-1 mice intranasally inoculated with WEEV.McM.FLUC and sacrificed at various time points. These animals were euthanized, decapitated, and whole heads were separated along the medial sagittal plane. Representative images are presented (Figure 2A–D) which illustrates the course of dissemination into the CNS. BLM signal was initially observed in the nasal turbinates and olfactory bulb. The infection proceeded along the lateral olfactory tract and ultimately progressed through CNS regions consistent with olfactory sense neuronal connectivity. Infection was invariably bilateral and intensified in regions consistent with basal nuclei, thalamus, and hypothalamus. Ultimately, FLUC expression was detected in neocortical regions and the brainstem by day 3 PI. Luciferase activity in the brainstem was separated into two distinct regions. The midbrain expression of FLUC appeared continuous with basal nuclei, thalamus, and hypothalamus and it was here that the greatest BLM was observed. BLM signal within the pons was discontinuous with signal from superior regions and failed to approach levels seen within basal nuclei, thalamus, hypothalamus, or cerebrum. Interestingly, the cerebellum was consistently spared from infection despite high BLM activity within posterior pons (cerebellum’s site of attachment).

### Characterization of Chemokine Induction Resulting from Infection with WEEV.McM.FLUC

Chemokines associated with severe CNS inflammation [46] and previously shown to be highly induced during McM infection [41] were measured in whole brain homogenates to compare the inflammatory responses within the CNS between WEEV.McM and WEEV.McM.FLUC (Figure 2E–F). Robust expression of both MCP-1 and IP-10 was observed in both infected groups of mice. Although WEEV.McM.FLUC was found to be attenuated when compared to wild-type McM in terms of PFU/g brain tissue (Figure 1F) and chemokine induction (Figure 2E&F), manifestation of clinical disease was comparable. This is supported by MTD (3.0 days vs. 3.1 days) and signs of disease exhibited in both groups. Early events indicated that inflammatory markers resulting from WEEV.McM.FLUC infection were significantly less than those observed in McM infected animals at 24 hpi, but levels rapidly approached that of McM by the following day.

### Clinical Signs

All infected mice (n = 10) showed variably severe clinical signs namely depression and motor deficits culminating at 48–72 hours post infection (PI). Most affected animals developed ataxia, with rhythmic raising and lowering of front limbs alternatively. Reduced stride length was visually observed in affected animals during voluntary movement. Animals in this intermediate stage of the disease did not appear to have visual impairment as they remained responsive to visual stimulation. In a later stage, animals were unresponsive to visual stimuli, but were typically responsive to touch. Mice showed unresponsiveness during handling only in the latest stage of disease (≥72 hpi). Lateral recumbency with tachypnea was characteristic of this terminal stage of the disease.

### Pathology and Immunohistochemistry

**Figure 3:**
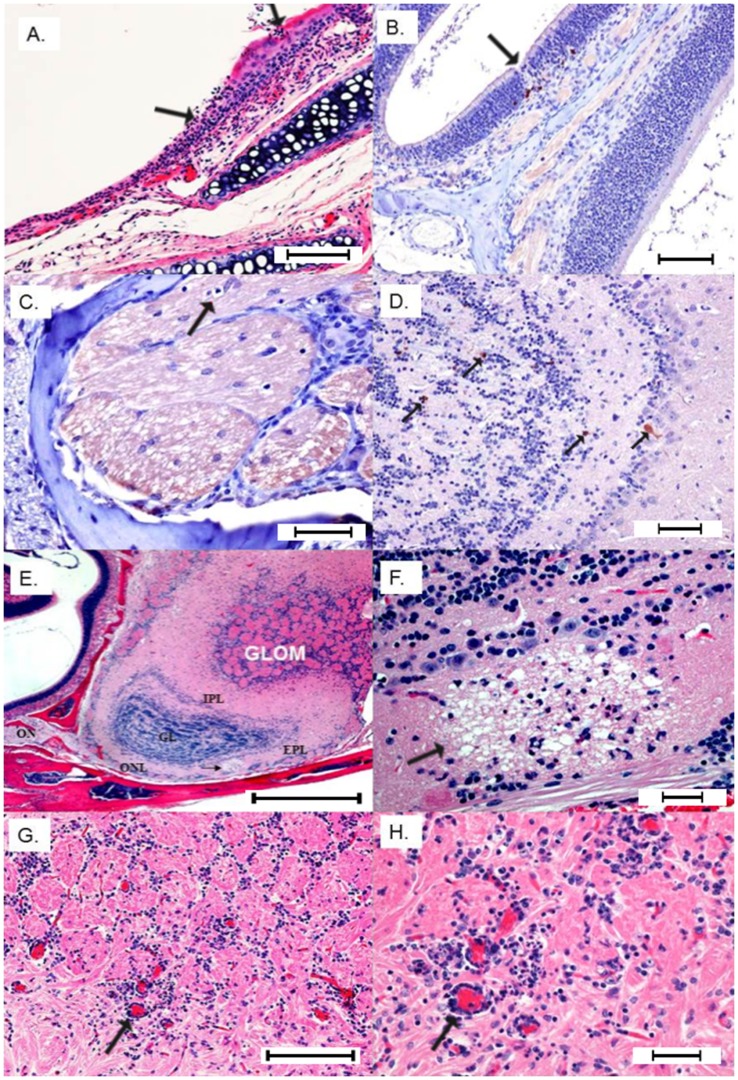
I. Extraneural lesions 24 hpi. A) Focal erosion/necrosis of the olfactory mucosa with deciliation of the flanking epithelium and neutrophil infiltration into the mucosa and submucosa (Bar = 100 µm). B) IHC positive staining for FLUC is highlighted in a few neuropeithelial cells subjacent to a focal loss of olfactory mucosa (Bar = 200 µm). **II- Neuroinvasion from olfactory nerve 48–72 hpi.** C) Terminal end of olfactory nerve shows a digestion chamber (arrow) with occasional lymphocytes infiltrating vacuolated branches (Bar = 100 µm). D) Early immunoreactivity (anti-FLUC) in the main olfactory bulb involving scattered neurons in the external plexiform and granular layers (Bar = 100 µm). E) Sagittal section H&E showing the connection between olfactory nerve (ON) and main olfactory bulb layers affected by multifocal necrotizing lesions with associated status spongiosis and infiltration of neutrophils. Glom = glomerular layer; EPI = external plexiform layer; IPL = internal plexiform layer; and ONL = olfactory nerve layer at the ventrum of the olfactory bulb (Bar = 400 µm). F) Neuropil of the olfactory nerve layer shows a large vacuolar lesion (demyelination) with individual neuronal necrosis and infiltration of small numbers of neutrophils (Bar = 100 µm). G) Perivascular cuffs and multifocal gliosis in the glomerular layer 72 hpi (Bar = 200 µm). H) Close-up view of the congested glomerular vessels with pleocellular perivascular cuffs comprising moderate numbers of neutrophils, lymphocytes and glial cells (Bar = 100 µm).

**Figure 4:**
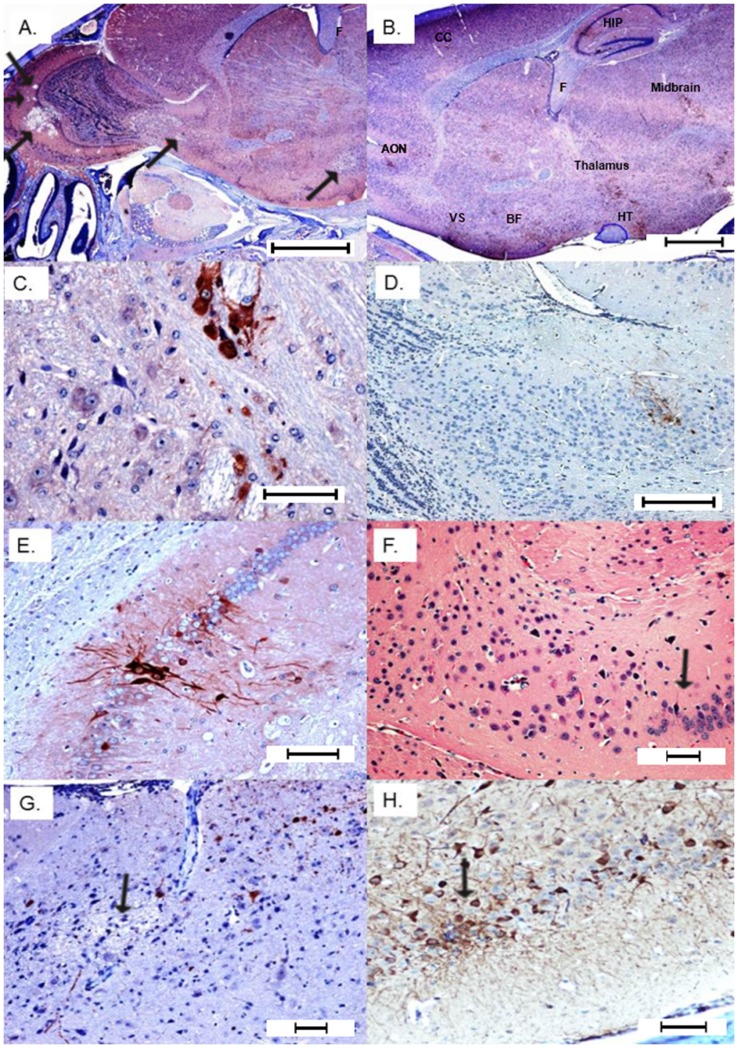
Later stage dissemination throughout the brain 72 hpi. A) Expanded view of the olfactory bulb showing progression of virus into caudal regions of the brain with multifocal necrosis and secondary demyelination (arrows) (Bar = 1000 µm). B) Multifocal demyelination and positive immunoreactivity in the anterior olfactory nucleus (AON), ventral striatum (VS), basal forebrain (BF) thalamus, hypothalamus, midbrain, hippocampus (HIP), and cerebral cortex (CC). Fornix (F) and optic tract (circled in blue) do not show any immunoreactivity (Bar = 1000 µm). C) Neuronal immunoreactivity in caudal olfactory bulb that shows multifocal demyelinating lesions (Bar = 100 µm). D) Multifocal immunoreactivity in caudal olfactory bulb and anterior olfactory nucleus (Bar = 200 µm). E) Strong immunoreactivity in hippocampus (Bar = 100 µm). F) Hippocampal H&E showing focal loss of pyramidal neurons with mild gliosis (Bar = 100 µm). G) Multifocal areas of necrosis and demylelination in the cerebral cortex (Bar = 200 µm). H) Strong immunoreactivity in cerebral neurons and their dendrites revealing interneuronal spread (Bar = 100 µm). All IHC images within this figure are from anti-FLUC staining. Comparison images using anti-WEEV staining may be found in the supplemental information.

**Figure 5:**
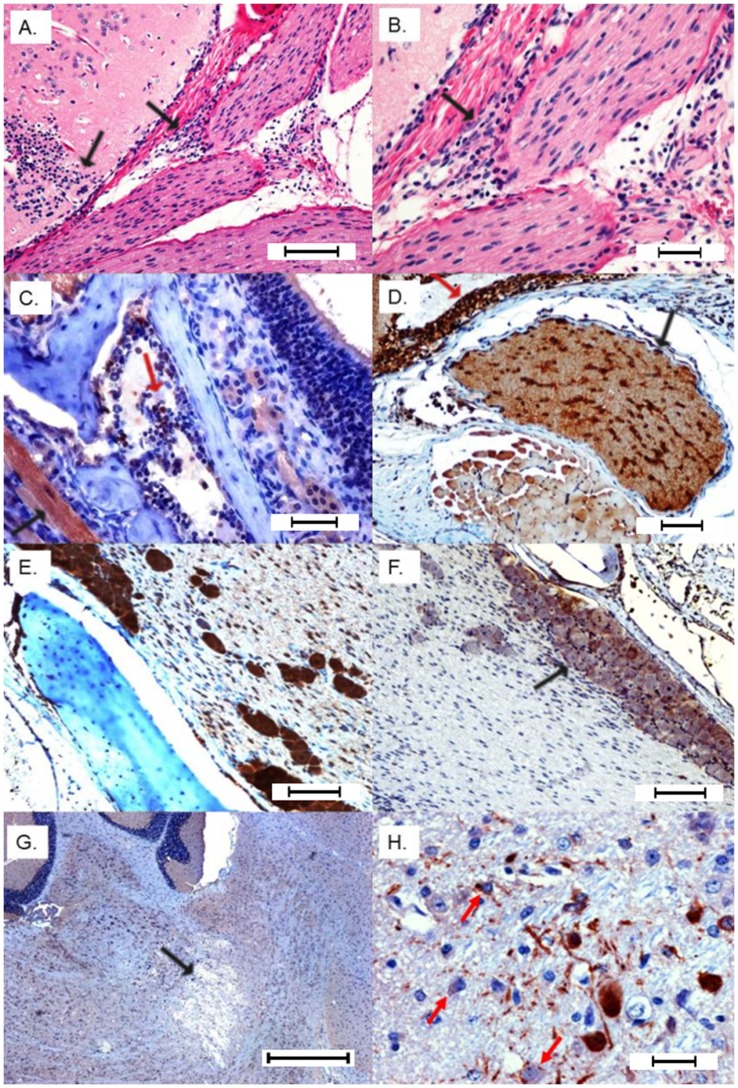
Neuroinvasion from trigeminal nerve. A) Cranial nerves including a branch of trigeminal nerve show neutrophilic perineuritis with a large glial nodule extending to the meninges of the overlying brain section (Bar = 200 µm). B) Close up of epineurium of cranial nerves infiltrated by neutrophils and lymphocytes (Bar = 100 µm). C & D) Strong immunoreactivity (anti-FLUC) of maxillary nerve including Schwann cells. Note strong and diffuse immunoreactivity of olfactory neuropeithelium, variable staining of surrounding skeletal muscles and bone marrow elements (Bar = 100 µm). E) Trigeminal ganglion IHC positivity (Bar = 100 µm). F) Trigeminal positivity is associated with immunoreactivity of the overlying meninges and brain tissue (Bar = 100 µm). G) Brainstem demyelinating lesion (potential consequence of trigeminal invasion) (Bar = 400 µm). H) IHC positivity (anti-FLUC) in the brain stem with interneuronal spread and rare immunoreactivity of glial cells (astrocytes) (Bar = 100 µm). All IHC images within this figure are from anti-FLUC staining. Comparison images using anti-WEEV staining may be found in the supplemental information.

Sagittal sections of the head were selected to facilitate viewing the nasal mucosa, olfactory nerve as it crosses the cribriform plate and connects to the bulb to help determine anatomic locations of the brain lesions (Figure 2 diagram). Pathologic lesions were observed in histological specimens prepared from imaged mice and in mice receiving WEEV.McM. WEEV.McM and WEEV.McM.FLUC produced comparable lesions. Serial sections were stained using immunohistochemical methods (anti-FLUC and anti-WEEV for recombinant virus while only anti-WEEV was used for wt virus infections) to demonstrate viral expression at the affected sites. Lesions and luciferase immunopositivity were observed to follow the same pattern as the imaged FLUC activity (Shown repeatedly in Figures 3, 4, and 5). IHC staining of both WEEV antigen and luciferase revealed that infection was almost exclusively limited to neurons and that dissemination was likely through the neuronal connectivity. Histopathologic alterations encountered within the nasal cavity and brain are summarized as follows:

#### Phase I: extraneural viral lesions

Twenty four hours PI: luminal aggregates of moderate numbers of neutrophils and fewer lymphocytes were detected in the nasal cavity with focal deciliation of respiratory mucosa corresponding to the areas of inflammation. In markedly immunopositive animals there was a focal erosion/ulceration (full thickness necrosis) of the respiratory mucosa and extension of the inflammatory exudate into the adjacent congested submucosa (Figure 3A). IHC (anti-FLUC) revealed immunoreactivity of variable numbers of neuroepithelium (Figure 3B). The numbers of positive cells increased with the severity of the clinical symptoms. The terminal end of the olfactory nerve before crossing the cribriform plate to merge into the olfactory bulb showed a mild degree of neuropathy with occasional digestion chambers indicative of Wallerian-type degeneration or secondary demyelination (Figure 3C), indicative of impaired axonal transport. Occasional lymphocytes were detected in the affected branches of the olfactory, maxillary, glossopharyngeal, and hypoglossal nerves.

#### Phase II: Neuroinvasion

24–48 h PI: In the olfactory bulb, immunoreactivity (anti-FLUC or anti-WEEV) showed high degree of neuronal specificity within CNS tissue (Figure 3D and Figure S2). Neuronal necrosis was commonly evident in the glomerular, granular, external plexiform and internal plexiform layers plus the olfactory nerve layer at the ventrum of the bulb. In the areas of microcavitation, there were infiltrations of a few neutrophils, glial cells and lymphocytes (Figure 3E–F). Perivascular cuffing was prominent throughout affected areas (Figure 3G–H). Also, myeloid cavities of the head showed variable immunoreactivity in lymphoid precursors and monoblasts. Surrounding skeletal muscles showed inconsistent immunoreactivity.

#### Phase III: CNS dissemination and associated lesions

48–72 h PI: The severity of the lesions in the olfactory bulb increased and the lesions started to propagate into more caudal regions of the brain (Figure 4). Multifocal areas of necrosis along with positive immunoreactivity were detected in the anterior olfactory nucleus, ventral striatum and basal forebrain at the ventrum of the brain. Dorsally, cerebral cortex was multifocally involved along with the pia matter and Virchow-Robin space (fluid-filled canals that surround perforating arteries and veins in the parenchyma of the brain). Other areas that were consistently involved were hippocampus, thalamus, hypothalamus, caudate putamen, mid brain, cerebellar superior peduncle, and pontomedullary region. Cranial nerves also showed a focal to multifocal immunoreactivity especially trigeminal nerve and its ganglia along with optic and cochlear nerves. Trigeminal pathways indicated significant immunopositivity and moderate to severe pathologic alterations namely chromatolysis, vacuolation and individual neuronal loss (Figure 5). To ensure that anti-FLUC staining was truly representative of viral localization, IHC staining of WEEV.McM and WEEV.McM.FLUC antigen was performed to discern any differences in viral distribution and localization to lesions. IHC staining with anti-WEEV polyclonal serum showed indistinguishable staining patterns and localization of the lesions (Figures S2 and S3) compared to anti-FLUC IHC staining. Additionally, anti-WEEV IHC revealed that sinus hairs (vibrissae) were affected, supporting trigeminal nerve pathway involvement (Figure S4).

### Antiviral Efficacy Testing

**Figure 6:**
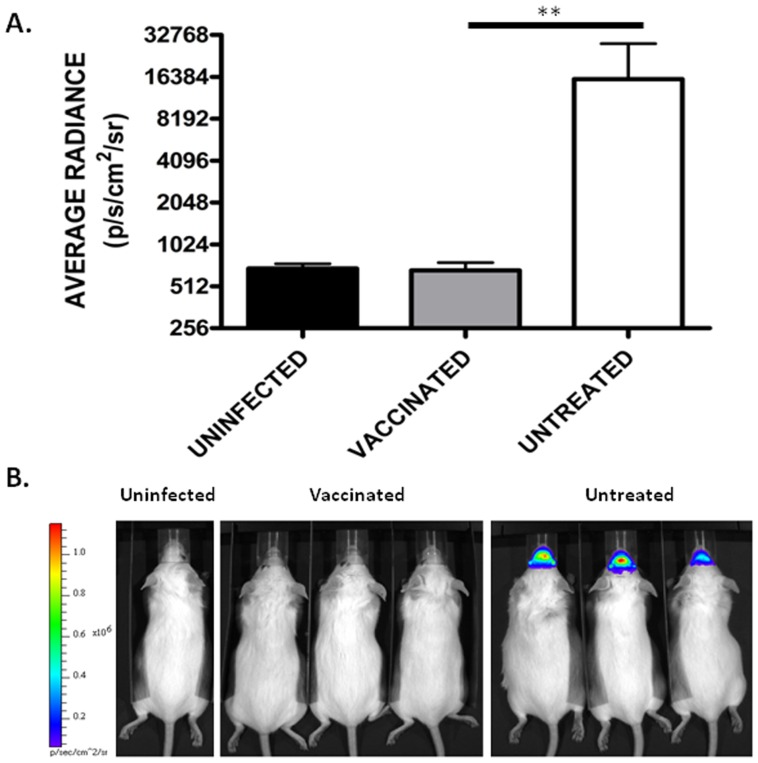
*In vivo* imaging assessment of liposome-antigen-nucleic acid-complex vaccination strategy. A) Average radiance in vaccinated, untreated, and uninfected mice at 48 hpi. Treated animals show significantly reduced luciferase activity (*p*<0.01) compared to untreated animals. Error bars indicate standard deviation. B) Corresponding images used for the quantitative analysis. Scale was normalized for all images and is shown on the left-hand side. Images presented are from 48 hours post intranasal challenge with WEEV.McM.FLUC.

To demonstrate the utility of *in vivo* imaging technology in the development of effective antivirals, mice 4–6 weeks old were vaccinated with CLNCs and WEEV E1 glycoprotein ectodomain using a prime-boost strategy. Prime-boost vaccination, with WEEV E1 glycoprotein ectodomain, results in 100% protection from challenge with 1×10^4^ PFU of WEEV.McM.FLUC via intranasal inoculation. Animals were imaged at 48 hpi (Figure 6B). Quantitative analysis on resulting images was performed and shows a significant reduction of bioluminescent signal in treated compared to untreated mice (*p*<0.01) (Figure 6A).

## Discussion

In this report, we have shown that a WEEV-based AES is capable of inducing lethal encephalitic disease in a mouse model. Optimal *in vivo* reporters such as firefly luciferase may be robustly expressed from recombinant virus despite their large coding sequence (1.6 kb). Immunohistochemistry and BLM measurements have shown that WEEV.McM.FLUC retains functional transgene expression throughout CNS dissemination when delivered by intranasal routes. BLM imaging of FLUC activity provided approximation of viral titer within the brain and gross-scale visualization of disease progression. While measurably attenuated in both viral replication kinetics and in the induction of immunological markers of disease, recombinant WEEV.McM.FLUC remained indistinguishable from wild-type virus in terms of histopathological lesions and MTD. This finding supports the use of AES in the assessment of antiviral strategies targeting aerosol exposure. Beyond approximating viral titers, convenient quantification of reporter signal may provide powerful inferences towards therapeutic efficacy and mechanism of protection. Therapeutics targeting viral replication, for example, should be capable of significantly decreasing reporter level and distribution. We show that vaccination with liposome-antigen-nucleic acid-complexes provides significant protection from challenge with WEEV.MCM.FLUC and that quantitative analysis of BLM does approximate prophylactic antiviral efficacy. As effective therapeutic strategies become available, BLM imaging would provide an excellent platform in which to rapidly evaluate such strategies.

*Ex vivo* imaging enhanced correlative histopathological examination as lesions here compared with viral expression levels immediately prior to euthanization. In the case of WEEV, CNS regions associated with viral expression indicated a preference for neuroinvasion through olfactory pathways. When examining the olfactory system, the initial connectivity can be characterized as a tremendous convergence of many olfactory sensory neuron (OSN) dendrites. In the rodent, an estimated 2000 olfactory bulb glomeruli are innervated by 5×10^6^ OSN. Each glomerulus possesses an estimated 75 mitral and tufted (MT) neurons that receive information from OSNs. This equates to roughly 1000 OSNs for every MT neuron [47], [48]. Therefore, infection of a proportion of OSNs may result in convergence of advancing infection.

The neuronal connectivity from the glomerulus is thought to extend into several brain regions. Unlike other sensory systems, the olfactory bulb may send its output directly to olfactory cortex without obligate processing through the thalamus [49] although thalamic pathways are also utilized. The connectivity of MT cells was recently detailed in an elegant study utilizing viral tracing techniques [50]. The authors of that study determined that MT cells synapse with a very large set of target neurons, to include neurons of the lateral olfactory tract, but also among other olfactory bulb neurons, including granule cell layer neurons near the mirror symmetric glomerulus (a feature not found in other sensory systems). More research is needed to determine what role the glomerular neuronal connectivity plays in WEEV dissemination. It is conceivable that this architecture would favor increased viral titers within the olfactory bulb and thus lead to more efficient spread into the rest of the CNS. Bilateral communication is also available by means of the anterior commissure. The extensive connectivity of olfactory systems, provide broad and bilateral dissemination potential within the brain proper.

The olfactory bulb contains specialized dendrodendritic synapses, in which vesicles are observed within presynaptic and postsynaptic membranes. As synaptic plasticity is dependent in part upon translational machinery present at the synapse, the process of learning and memory may be intimately tied to encephalitic alphavirus infection. Interestingly, aside from general somatosensation the trigeminal nerve sends fibers to the neuroepithelium (to detect caustic stimuli). It is conceivable that the observed infection of trigeminal ganglia and brainstem could have originated from infected neuroepithelia. Such an alternative neuroinvasion mechanism has been reported for VEEV infected animals after ablation of olfactory bulbs [51]. CNS infection was associated with trigeminal nerve involvement. The current study shows a similar neuroinvasion for WEEV including a novel finding of virus localization to the follicular epithelium of vibrissae. Information from vibrissae is delivered via trigeminal nerve first into trigeminal sensory complex of the brain stem. From there the virus spreads to parts of the thalamus and barrel cortex, the most studied pathways from trigeminus to the cortex. WEEV is a naturally-occurring recombinant virus and resembles VEEV in its ability to infect trigeminal nerve-associated neurons and ultimately infect brainstem nuclei. Therefore, WEEV may serve as a relevant model system for higher priority pathogens such as VEEV or EEEV.

Studies aimed at examining WEEV neuroinvasion or CNS dissemination in the animal model should benefit from the use of bioluminescent imaging. There are no specific antivirals available for alphaviral infection and treatment is limited to supportive care. Studies have demonstrated the limitations of immunoprophylaxis [52], and while modulators of innate immunity show promise [41], [53], future generations of therapeutics may benefit from a greater understanding of the progression of alphaviral-induced neural disease. BLM may be useful to test antivirals, prophylactic treatments, and evaluate pathogenesis.

## Supporting Information

Figure S1 **Diagram of odorant-sensing tissues of the mouse.** The Gruenenberg ganglion is thought to be responsible for detecting odorants involved in suckling and these neurons synapse at the accessory olfactory bulb. The vomeronasal organ is responsible for detecting pheromones and also synapses at the accessory olfactory bulb. The olfactory sensory neurons within the olfactory epithelium synapse at the main olfactory bulb.
(TIF)

Figure S2 **Anti-WEEV in olfactory bulb.** Black arrow shows immunopositivity in the olfactory bulb at 72 HPI. Red arrows show immunopositivity in cortical and lateral olfactory tract.
(JPG)

Figure S3 **Anti-WEEV in the brainstem at 72 HPI.** Black arrows showing immunopositivity at site of a large demyelinating lesion (rarefaction of neuropil). Red arrows show additional immunopositivity throughout brainstem.
(JPG)

Figure S4 **WEEV antigen in the sinus hairs at 72 HPI.** Red arrow shows markedly immunopositive sinus hair. Black arrow shows adjacent sinus hair with milder immunoposivity.
(JPG)

Methods S1 **Detailed description of the molecular cloning events leading to the construction of WEEV.McM.FLUC recombinant virus.**
(DOCX)
