# Supplementary figures and images for: Bioluminescent Imaging and Histopathologic Characterization of WEEV Neuroinvasion in Outbred CD-1 Mice
Source: PLoS One. 2013 Jan 2;8(1):e53462. doi: 10.1371/journal.pone.0053462 (PMC3534643; doi:10.1371/journal.pone.0053462)

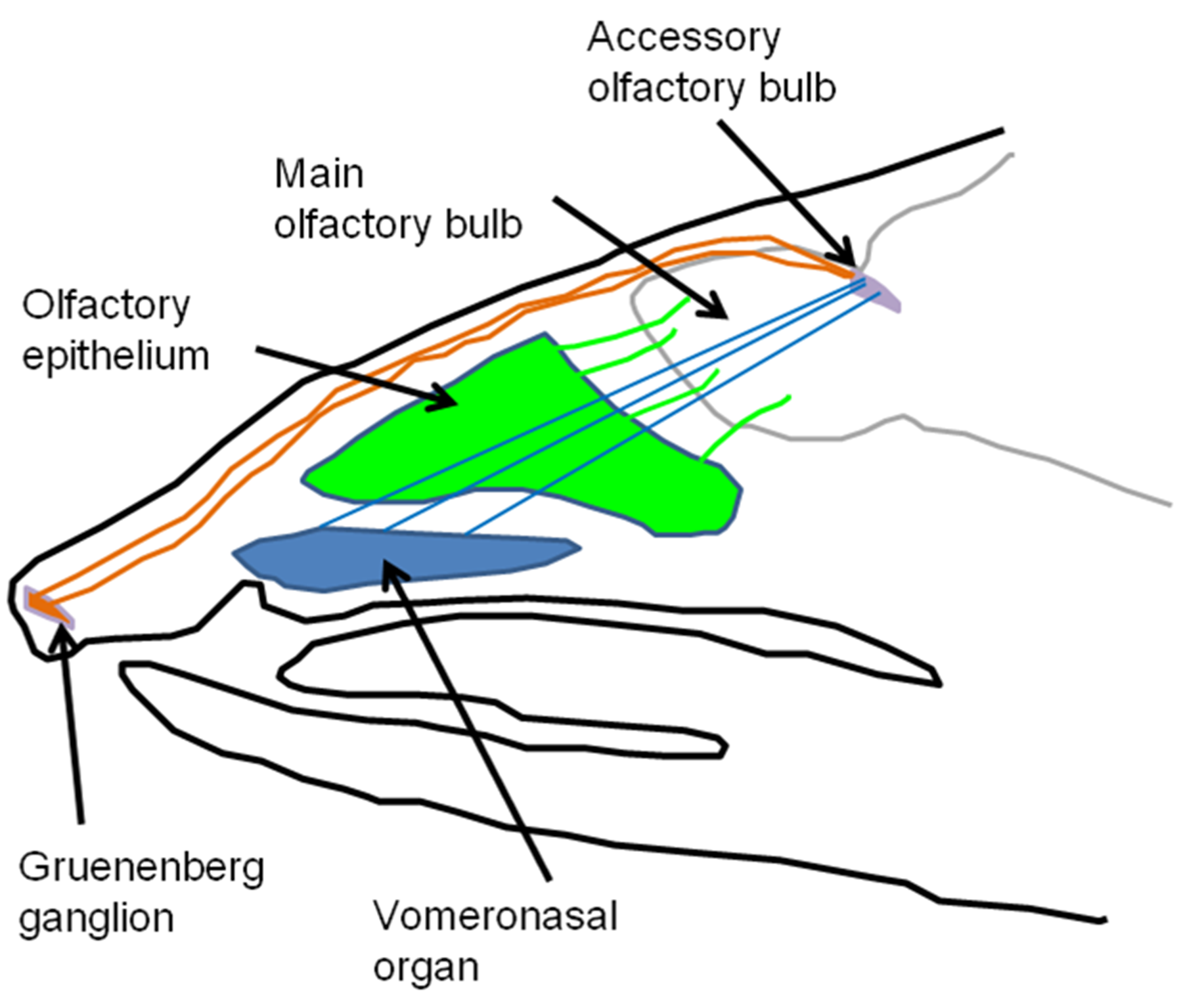

Supplement: Figure S1 — Diagram of odorant-sensing tissues of the mouse. The Gruenenberg ganglion is thought to be responsible for detecting odorants involved in suckling and these neurons synapse at the accessory olfactory bulb. The vomeronasal organ is responsible for detecting pheromones and also synapses at the accessory olfactory bulb. The olfactory sensory neurons within the olfactory epithelium synapse at the main olfactory bulb. (TIF) [file pone.0053462.s001.tif]

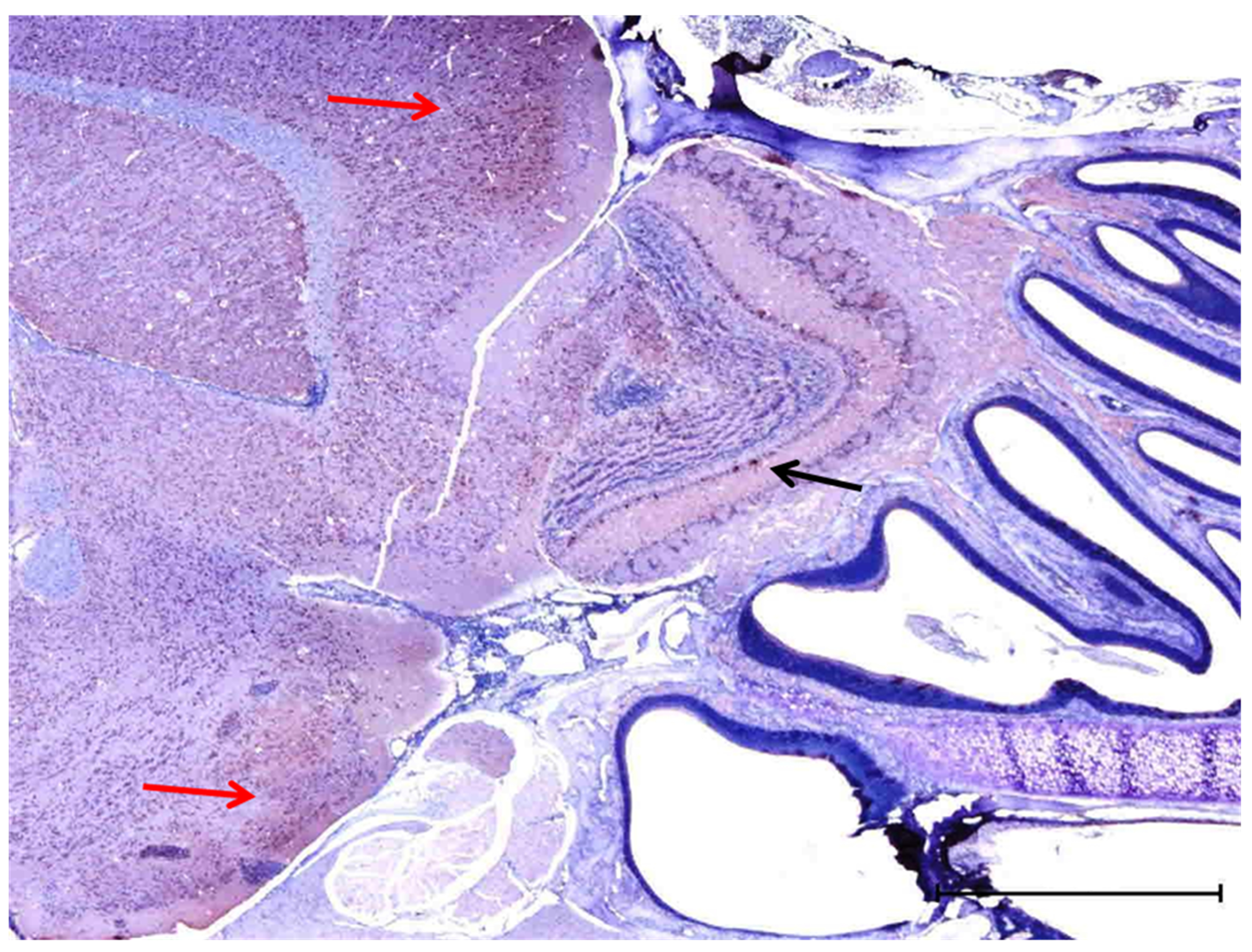

Supplement: Figure S2 — Anti-WEEV in olfactory bulb. Black arrow shows immunopositivity in the olfactory bulb at 72 HPI. Red arrows show immunopositivity in cortical and lateral olfactory tract. (JPG) [file pone.0053462.s002.jpg]

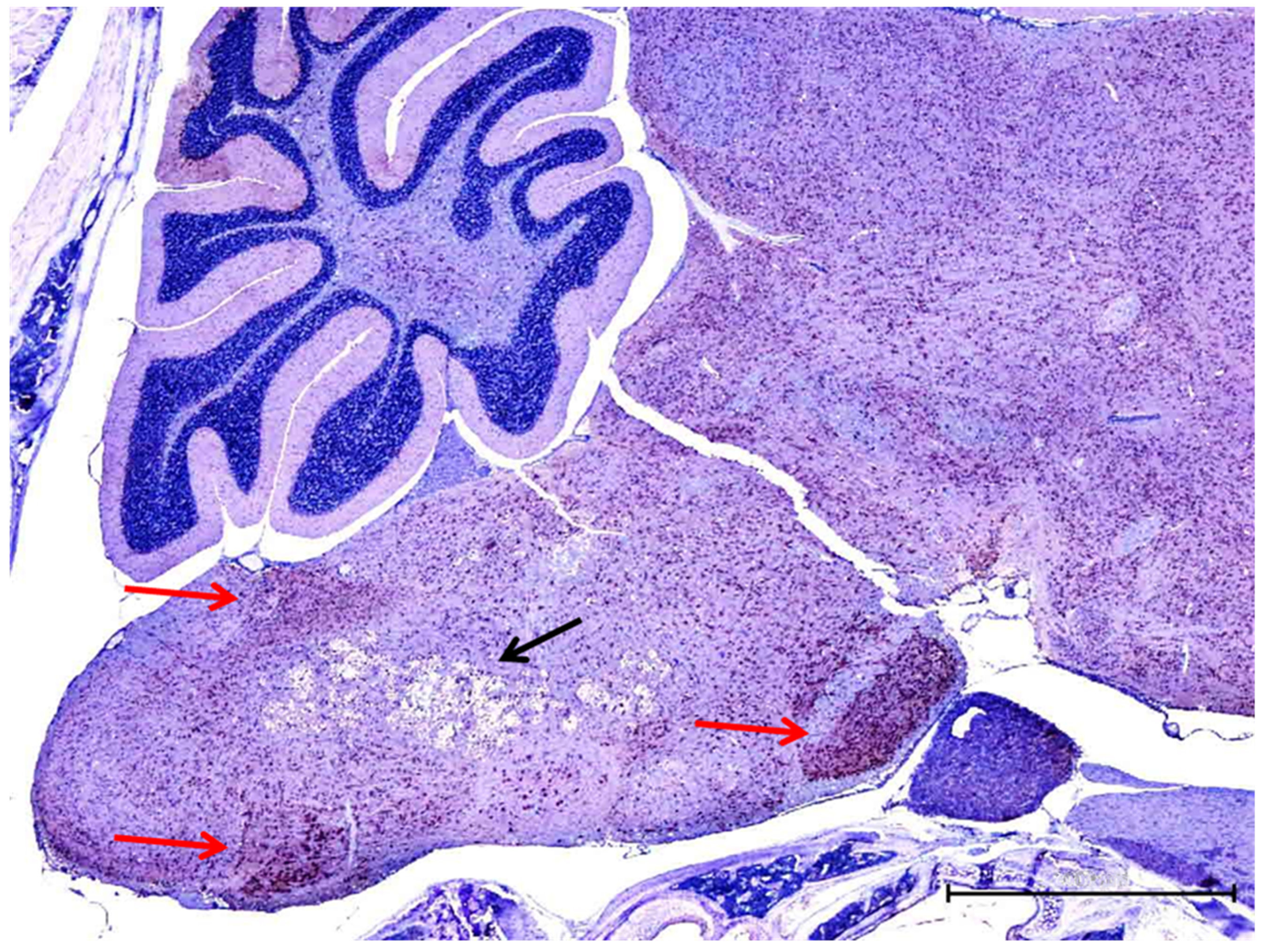

Supplement: Figure S3 — Anti-WEEV in the brainstem at 72 HPI. Black arrows showing immunopositivity at site of a large demyelinating lesion (rarefaction of neuropil). Red arrows show additional immunopositivity throughout brainstem. (JPG) [file pone.0053462.s003.jpg]

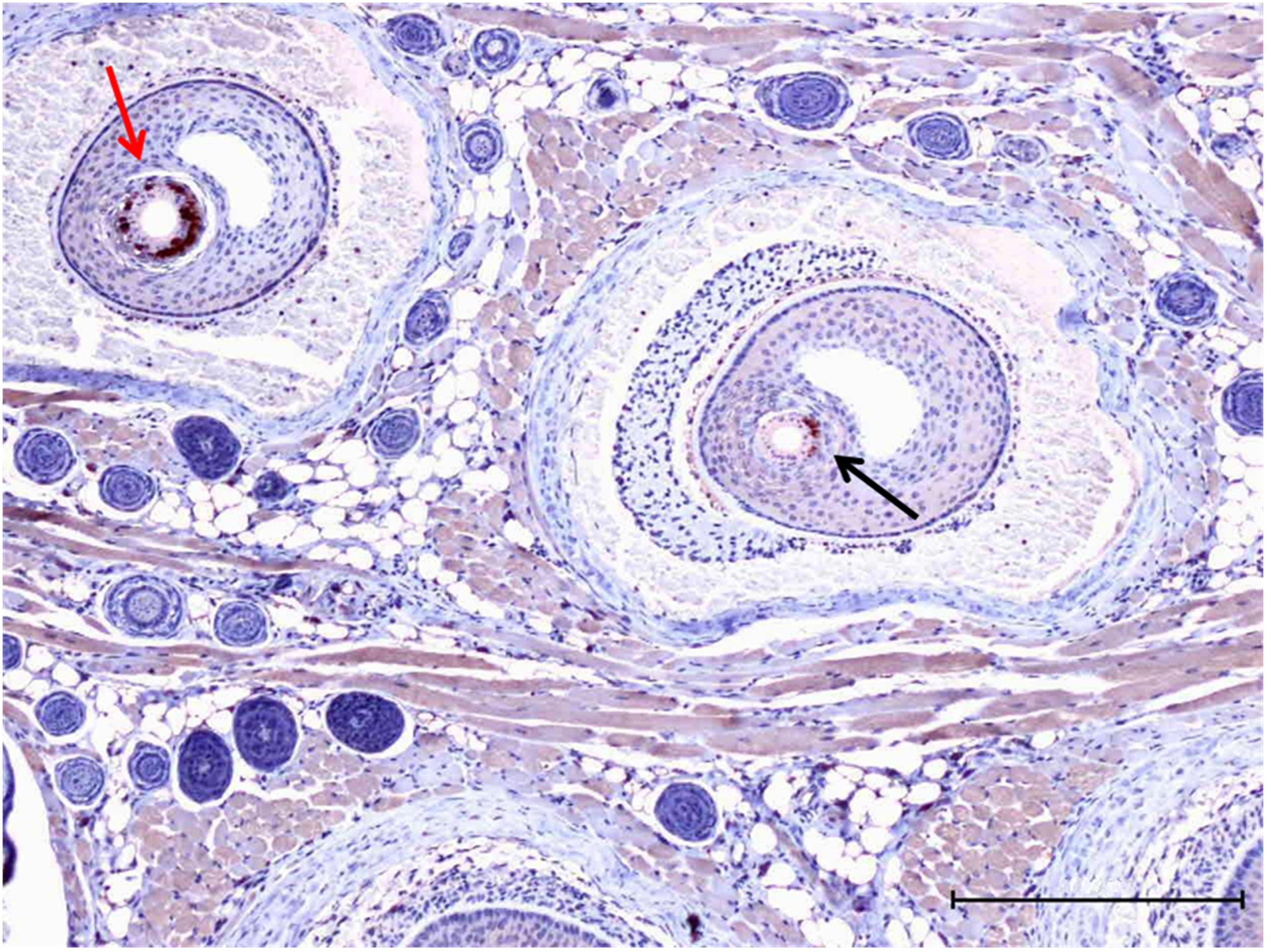

Supplement: Figure S4 — WEEV antigen in the sinus hairs at 72 HPI. Red arrow shows markedly immunopositive sinus hair. Black arrow shows adjacent sinus hair with milder immunoposivity. (JPG) [file pone.0053462.s004.jpg]
