# Supplementary material for: Bioluminescent Imaging and Histopathologic Characterization of WEEV Neuroinvasion in Outbred CD-1 Mice
Source: PLoS One. 2013 Jan 2;8(1):e53462. doi: 10.1371/journal.pone.0053462 (PMC3534643; doi:10.1371/journal.pone.0053462)
Supplement: Methods S1 — Detailed description of the molecular cloning events leading to the construction of WEEV.McM.FLUC recombinant virus. (DOCX) [file pone.0053462.s005.docx]

**Supporting Information**

**Methods S1**

**Virus construction**

A PCR fragment (fragment #1) was generated using the following primers (GCA CCG AAC GCA ACG GTA CCC ACA GCA TTA GC and CTG ACC GGT GCT CTT CGT CAT CTA CGT GTG TTT ATA AGC ATA GAG CTG CAG ACC AAC ACT ATA AGT CCA) from pMcM. A second PCR fragment (fragment #2) was generated from pMcM using the following primers (CAG ACC GGT CT GAG CGC GGC CAC TGA CAT AGC GGT AAA ACT CGA TGT ACT TC and CAG TCT AGA AAT ATT ATT GAA GCA TTT ATC AGG GTT ATT GTC TCA TGA GCG GAT AC). Fragment #1 was digested with KpnI and AgeI while fragment #2 was digested with AgeI and XbaI. The resulting digested fragments were gel purified and inserted (via two-part ligation) into a modified pUC19 plasmid which had the SapI sites removed via site-directed mutagenesis and which had been digested with KpnI and XbaI. The resulting intermediate plasmid was digested with SapI and AgeI. The following commercially synthesized oligonucleotide pairs were mixed in equal molar amounts, heated to 90º C, and allowed to cool slowly and anneal (CCA GGC TCT TCG TGA TCC AGA TAC GAG ATC ATA CTG GCA GGC CTG ATC ATC ACG TCC CTT TCC ACG TTA GCC GAA AGC GTT AAG AAC TTC AAG AGC ATA AGA GGG AAC CCA ATC ACC CTC TAC GGC TGA CCT AAA TAG GTG ACG TAG TAG ACA CGC ACC TAC CCA CCG CCA AAA GGC CGG CCA CCG GTG ACC; GGT CAC CGG TGG CCG GCC TTT TGG CGG TGG GTA GGT GCG TGT CTA CTA CGT CAC CTA TTT AGG TCA GCC GTA GAG GGT GAT TGG GTT CCC TCT TAT GCT CTT GAA GTT CTT AAC GCT TTC GGC TAA CGT GGA AAG GGA CGT GAT GAT CAG GCC TGC CAG TAT GAT CTC GTA TCT GGA TCA CGA AGA GCC TGG). The annealed double stranded oligonucleotides were also digested with SapI and AgeI. The digested products were ligated to form a new intermediate plasmid which contains a duplication of the subgenomic promoter immediately after the last nucleotide of E1. Following the duplicated subgenomic promoter is the engineered FseI and AgeI sites. These two sites act as the clone in site for transgene expression from the final construct. Firefly luciferase was cloned into the Age1 and FseI sites of the intermediate plasmid by engineering FseI and AgeI sites at the 5’ and 3’ ends (respectively) of the PCR fragment generated from pGL3 (Promega). Once transgene was determined to be appropriately inserted and accurate in terms of sequence, the intermediate plasmid was digested with KpnI and MfeI, gel purified, and ligated into the KpnI and MfeI digested and dephosphorylated full-length infectious clone. Final plasmids were sequenced to validate proper insertion orientation.
